# Patterns of Tumor Infiltrating Lymphocytes in Adenoid Cystic Carcinoma of the Head and Neck

**DOI:** 10.3390/cancers14061383

**Published:** 2022-03-08

**Authors:** Johannes Doescher, Moritz Meyer, Christoph Arolt, Alexander Quaas, Jens Peter Klußmann, Philipp Wolber, Agnes Bankfalvi, Hans-Ulrich Schildhaus, Tobias Bastian, Stephan Lang, Simon Laban, Patrick J. Schuler, Cornelia Brunner, Thomas K. Hoffmann, Stephanie E. Weissinger

**Affiliations:** 1 Department of Otorhinolaryngology, Head and Neck Surgery, University Hospital Ulm, 89075 Ulm, Germany; simon.laban@uniklinik-ulm.de (S.L.); patrick.schuler@uniklinik-ulm.de (P.J.S.); cornelia.brunner@uniklinik-ulm.de (C.B.); t.hoffmann@uniklinik-ulm.de (T.K.H.); 2 Department of Otorhinolaryngology, Head and Neck Surgery, University Hospital Essen, University of Duisburg-Essen, 45147 Essen, Germany; moritz.meyer@uk-essen.de (M.M.); tobias.bastian@uk-essen.de (T.B.); stephan.lang@uk-essen.de (S.L.); 3 Department of Pathology, Medical Faculty, University of Cologne, 50973 Cologne, Germany; christoph.arolt@uk-koeln.de (C.A.); alexander.quaas@uk-koeln.de (A.Q.); 4 Department of Otorhinolaryngology, Head and Neck Surgery, Medical Faculty, University of Cologne, 50924 Cologne, Germany; jens.klussmann@uk-koeln.de (J.P.K.); philipp.wolber@uk-koeln.de (P.W.); 5 Institute of Pathology, University Hospital Essen, University of Duisburg-Essen, 45147 Essen, Germany; agnes.bankfalvi@uk-essen.de (A.B.); hans-ulrich.schildhaus@uk-essen.de (H.-U.S.); 6 Institute of Pathology, University Hospital Ulm, 89081 Ulm, Germany; stephanie.weissinger@af-k.de

**Keywords:** adenoid cystic carcinoma, head and neck, tumor-infiltrating lymphocytes, tertiary lymphoid structures

## Abstract

**Simple Summary:**

Adenoid cystic carcinoma (ACC) is a rare tumor with late occurring metastases and recurrences, which makes it necessary for patients to be monitored closely even beyond the usual five years. So far, there is hardly any effective treatment in the palliative situation and trials on immunotherapeutic drugs have not been successful. We sought to find possible prognostic markers by analyzing patterns of tumor infiltrating immune cells and additionally to learn more about possible reasons for this lack of response to immunotherapy. It appears from our data that it is not relevant for prognosis if the tumor is infiltrated by immune cells. This might also be the reason that immunotherapies do not work in this particular disease, which suggests that ACC is not recognized by infiltrating immune cells. Therefore, the tumor would have to be made visible to the immune system, for example, through vaccinations.

**Abstract:**

Adenoid cystic carcinoma (ACC) is a rare malignancy in the head and neck. The prognosis remains poor and late recurrences often occur after 5 years and later. To date, there are no reliable prognostic markers for ACC. In several solid tumors, tertiary lymphoid structures (TLS) are associated with improved survival. This study aims to investigate the role of distribution patterns of tumor infiltrating immune cells (TIL) in ACC. A cohort of 50 patients from three different cancer centers was available for analysis. Sections were stained for CD3, CD4, CD8 and CD20 and evaluated with regard to their distribution of TIL. Patterns were determined as infiltrated-excluded, infiltrated-inflamed and presence of tertiary lymphoid structures. About half of the cases showed an infiltrated-excluded TIL pattern and only a minority of six cases had TLS present within the tumor. Within the inflamed phenotype CD3+ cells were by far the most abundant lymphocyte subtype, and within this compartment, CD8+ T cells were predominant. There was no influence on overall or disease-free survival by any of the TIL patterns. This indicates that ACC is a tumor with very low immunogenicity and even abundance of lymphocytes does not seem to improve prognosis for this disease. Therefore, the observed lack of response towards immunotherapy is not surprising and other methods to induce recognition of ACC by the immune system must be found.

## 1. Introduction

Adenoid cystic carcinoma (ACC) of the head and neck is a rare malignancy accounting for approx. 1% of all head and neck cancers. They arise mainly in the salivary glands, but also in the oral cavity and the paranasal sinus [1]. Treatment consists of surgery aiming for clear tumor margins and adjuvant radiotherapy (RT), which effectively prevents local recurrence [2]. However, the beneficial effect of RT on overall survival could not be proven in a larger analysis of data from the National Cancer Institute’s Surveillance, Epidemiology, and End Results (SEER) program [3]. This is mainly due to the development of distant metastases, even without local recurrence, which is reported in 19–52% of cases and can occur more than five years after initial diagnosis, which makes a close follow-up necessary, even after five years [4,5]. On a molecular level, especially expression of Programmed Death Ligand 2 (PD-L2) has been identified as a marker for worse prognosis and development of recurrences [6,7]. A comprehensive RNA expression analysis found a link between low T cell gene signatures and recurrence [8]. Another study, examining the tumor microenvironment (TME) immunohistochemically, made a similar observation with a low CD8+ T cell density being associated with a higher recurrence rate [9]. However, no precise analysis focusing on established spatial patterns of tumor infiltrating lymphocytes (TIL) including T helper cells and B cells has been completed, so far.

The high rate of distant metastases has put a lot of pressure on identifying effective systemic therapies, but with very limited success (reviewed in [10]). Especially, the use of immune checkpoint inhibitors (ICI) for ACC has not been able to reflect the success of these drugs in other entities, yet [11,12,13,14]. Most authors of these studies account for the low PD-L1 expression and low rate of CD8+ TIL in ACC tumor tissue for the failure of ICI [15]. However, this might not be the only reason and there are reports from other entities with response to ICI despite low or absent PD-L1 expression. Further, it seems to be important to have a reservoir of still functional or early dysfunctional T cells in close proximity to the tumor, which can be activated and respectively rendered functional by immune therapies [16].

These questions and the high rate and late development of metastases make it necessary to understand more about the TME of ACC. Moreover, this might add to understanding failure of the application of immunotherapy. Finally, biomarkers identifying patients at risk for late recurrence are needed to tailor follow-up strategies. As tertiary lymphoid structures (TLS) have been described as driving anti-cancer immunity and were found to be associated with improved outcome [17], we sought to evaluate the presence and clinical impact of TLS in ACC alongside with a spatial investigation of TIL patterns in the TME.

## 2. Materials and Methods

### 2.1. Patient Samples

Patients suitable for analysis were selected by a confirmed diagnosis of ACC and availability of archived tissue material from either a biopsy or surgical resection specimen at the time of initial diagnosis and treatment. Patients with material from recurrence or metastatic disease only were excluded. Optimal blocks with representative tumor areas were selected by the local pathologist at the respective study site. Clinical data comprised tumor staging, details on the treatment, age at diagnosis, last follow-up, information regarding recurrence and any reported deaths. The study was endorsed by the local ethics committee (no. 374/13) and the institutional review board.

### 2.2. Immunohistochemistry

FFPE sections were stained with HE. For the identification of cytotoxic T cells (CTL), CD3 and CD8 were applied. Furthermore, to define T helper cells, the sections were also stained for CD4, whereas CD20 was used to identify B cells and to evaluate tertiary lymphoid structures (TLS), in combination with CD3 (antibody details are listed in Table S1). Sections were stained on a DAKO Omnis GV900 autostainer (Agilent Technologies, Santa Clara, CA, USA). Pretreatment of the FFPE slides was performed with EnVision™ FLEX Target Retrieval Solution (Agilent Technologies, Santa Clara, CA, USA) and incubation lasted for 60 min. EnVision™ FLEX mouse or rabbit linker was used as secondary antibody and EnVision™ FLEX HRP Magenta for chromogen staining (both Agilent Technologies, Santa Clara, CA, USA).

**Figure 1:**
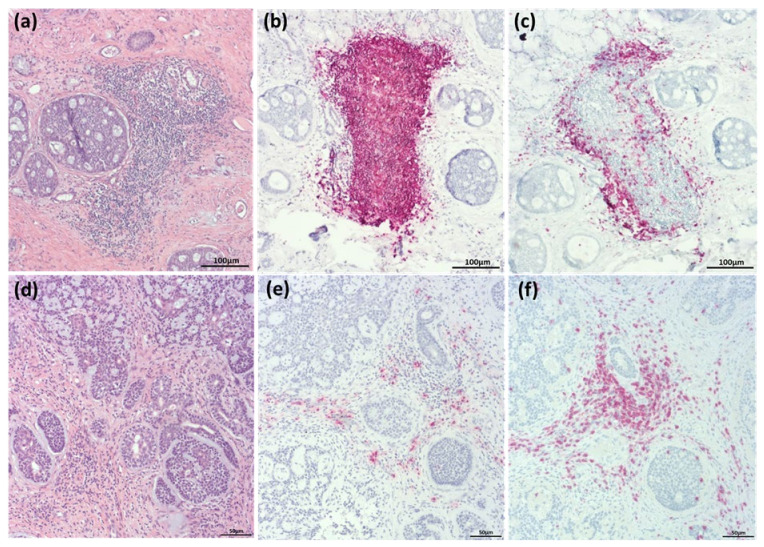
Examples of ACC of the submandibular gland (**a**) (HE) and the nasal cavity (**d**) (HE), categorized into infiltrated-inflamed with TLS (**a**–**c**) and infiltrated-inflamed without TLS (**d**,**e**). Corresponding immuno-histochemistry for CD20 is shown in (**b**,**e**), for CD3 in (**c**,**f**).

Evaluation was completed with regard for the distribution of tumor infiltrating lymphocytes (TIL), according to a previously published classification system [18,19]. In this, recurrent patterns of distribution of the immunologic tumor micromilieu are described. Basically, these patterns can be divided into three categories: I. I-E, “infiltrated-excluded”, CTLs are present in regions of the tumor borders, but not in the tumor core. II. I-I “infiltrated-inflamed”, numerous CTL can be detected in the tumor core and III. I-I (TLS): a special form of the I-I pattern, additionally showing the formation of so-called tertiary lymphoid structures (TLS), characterized by an accumulation of T- and B-cells and resembling a lymphoid structure, in direct proximity to the tumor (examples are shown in Figure 1).

### 2.3. Image Processing

Images were obtained using a Carl Zeiss Axioskop 2 microscope with a Zeiss Axiocam 105 color digital camera and 5×/0.515 and 10×/0.30 Plan Neofluar objectives with corresponding software (ZEN 3.1 blue edition). Image processing was performed with Microsoft PowerPoint, Version 2020.

### 2.4. Statistical Analysis

All statistical computations were performed with SPSS v28 (IBM, Armonk, NY, USA). Differences regarding the percentage of immune cell subsets were assessed using a two-tailed Mann–Whitney test. For comparisons between groups, a Chi^2^ test or, if necessary, a Fisher’s Exact test was applied. Figures were prepared with Prism 9 (Graphpad, San Diego, CA, USA). The venn diagram was created with the open-source software jvenn [20]. Statistical significance was assumed for *p*-values < 0.05.

## 3. Results

### 3.1. Clinical Parameters

**Figure 2:**
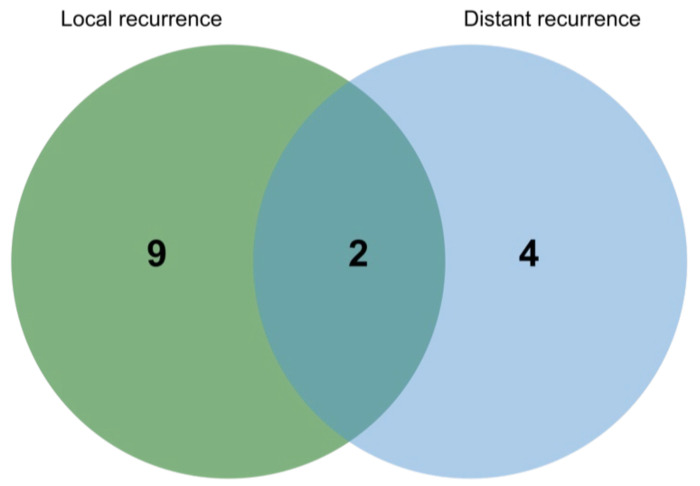
Distribution of recurrences without distant metastases at initial diagnosis.

The cohort consisted of a total of 50 patients from three different head and neck centers (Ulm: *n* = 32, Essen: *n* = 10, Cologne: *n* = 8). The mean age at diagnosis was 57.7 years, and slightly more patients were female (54%). Most tumors arouse in the submandibular gland (34%), followed by the parotid gland (18%) and the paranasal sinus (12%). Distribution of early vs. advanced T stage was fairly even with 54% T1–T2 and 46% T3–T4 stage. Most patients had no lymph node involvement (76%) and 16.3% presented with distant metastasis at the time of first diagnosis. Perineural spread was seen in 53.5% of patients treated with surgery. Treatment regimen for the primary disease consisted of surgery alone (34%), surgery with adjuvant RT (54%) and RT alone (10%). No information on treatment could be obtained for one patient (2%). Failure was observed in a total of 15 patients (30%) with the majority experiencing local failure alone (18%) and 12% developing distant metastases with or without local failure, 4% and 8%, respectively (Figure 2). Local failure was seen in 3 patients with surgery only, in 1 patient with RT alone and in 5 patients with surgery followed by RT. Distant failure occurred only in patients treated with surgery and combined local and distant failure in 1 patient after multimodal treatment and 1 patient after RT alone. The mean follow-up time was 5.6 years with a standard deviation of 4.1 years. 26 patients could be followed for more than 5 years, of which 9 for even more than 10 years.

### 3.2. Analysis of TIL Subtypes

**Figure 3:**
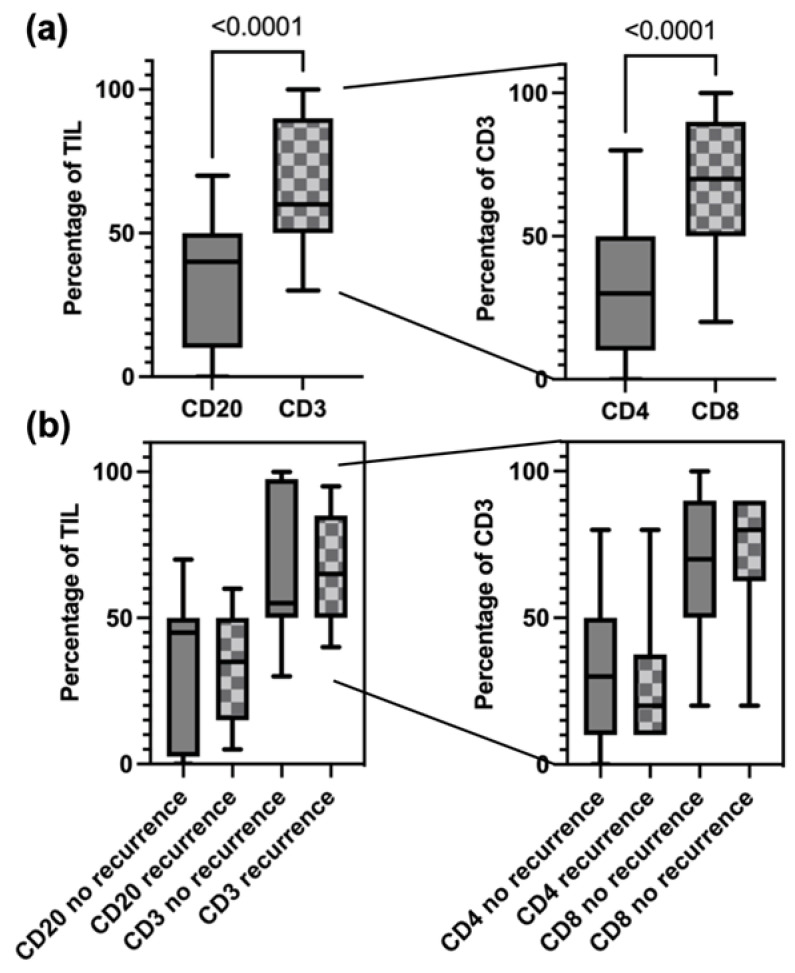
Distribution of TIL subtypes in the whole cohort (**a**) and with respect to recurrence (**b**).

In order to classify the cases into the three categories I-I, I-I (TLS) and I-E, as mentioned above, we looked for different types of lymphocytes, i.e., CD3+, CD4+, CD8+ and CD20+ cells. There was a relevant number of tumors (*n* = 22) which were not infiltrated or infiltrated by only a few single immune cells. Further analysis of infiltrating subtypes was therefore focused on tumors with sufficient TIL counts in the tumor core. Within TIL, there was a significant dominance present for CD3+ T cells as compared to CD20+ B cells (*p* < 0.0001). Within the CD3+ compartment significantly more cells were CD8+ than CD4+ (*p* < 0.0001) as shown in Figure 3a. Next, we sought to analyze if the composition of TIL was associated with the development of recurrence but could not detect any significant difference between the patients with a recurrence and those without (Figure 3b).

### 3.3. Correlation of TIL Patterns and Clinical Parameter

As described above, TIL patterns were categorized into infiltrated-excluded (I-E), infiltrated-inflamed (I-I) and presence of TLS (examples in Figure 1). There were 22 patients falling into the category of I-E (44%), 28 patients with an I-I pattern (56%) and TLS were present in specimens of 6 cases within this group. When correlating the first two patterns to clinical parameters, only the choice of therapy was significantly associated with the TIL pattern. Patients with an I-E pattern were more likely to receive surgery only whereas more patients with an I-I pattern were treated with surgery and adjuvant RT (*p* = 0.03). As therapy is recommended according to tumor stage, this pattern should have been reflected in the TNM staging. It can be stated that I-E patients tended to present with lower stages than I-I patients and a trend towards this could be observed for the M category with a higher proportion of patients with metastatic disease in the I-I group (*p* = 0.06). When looking at the overall stage groups, a significant association between very advanced disease (stage IVB and IVC) and the I-I pattern could be seen. Earlier disease stages were almost evenly distributed to one of the two groups, but 10 out of 12 stage IVB or IVC tumors showed an I-I pattern (*p* = 0.03). All correlations to clinical parameters can be found in Table 1.

### 3.4. Analysis of TIL Patterns with Regards to Recurrence and Metastasis

Following the correlation of the spatial TIL distribution patterns, we next analyzed a possible influence on recurrence. As shown in Figure 2, a total of 14 recurrences occurred in the follow-up period. Recurrence was fairly evenly distributed between the two patterns with 46.7% of recurrences developing from an I-I primary tumor and 53.3% from an I-E primary tumor. Within all recurrences, I-E tumors tended to develop more distant metastasis and local recurrences were observed more in the I-I group, although without reaching statistical significance. In an initial analysis, only the Ulm cohort (*n* = 32) was interrogated. Here, we found TLS to be associated with a better outcome in terms of recurrence. However, when incorporating samples from the two other centers to increase the sample size and trying to verify this observation, it could not be maintained as there were very few cases with a presence of TLS on the one hand and two cases with TLS developed a recurrence on the other hand. Additionally, one case with TLS presented with a distant metastasis at primary diagnosis (Table 2). TLS were further analyzed with regards to the presence of germinal centers and accumulation of high endothelial venules (HEV). Out of six cases, two showed presence of germinal centers and HEV at different densities (Table S2). Of note, these were the two cases encountering recurrence, but without reaching statistical significance due to the small number of TLS+ cases (*p* = 0.07).

To gain a deeper understanding of how the distribution of TIL might influence the outcome, we calculated a CD4/CD8 ratio for tumors with a measurable immune infiltrate (I-I) and dichotomized into a ratio of <1 and ≥1. In patients, where an immune infiltrate was seen, a total of 23 cases showed a low ratio (69.7%). The majority of recurrences was observed for those with a CD4/CD8 ratio below 1 (9 of 10 cases). In addition, the majority of the cases with distant metastases at primary diagnosis showed a low CD4/CD8 ratio. Again, this was without reaching statistical significance.

### 3.5. Survival Analysis

**Figure 4:**
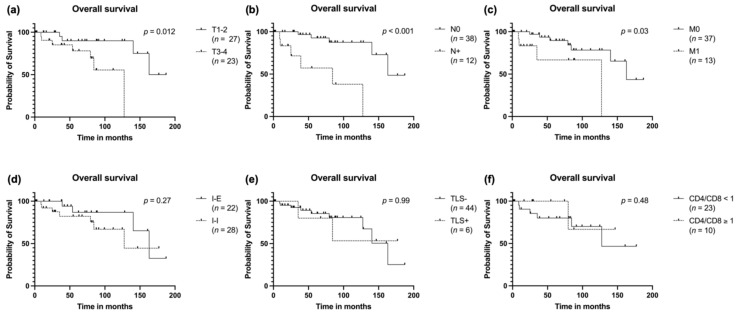
Overall survival stratified to (**a**) T stage, (**b**) N stage, (**c**) distant metastasis, (**d**) spatial distribution of TIL, (**e**) presence of TLS in the primary tumor and (**f**) CD4/CD8 T cell ratio.

Survival analysis was completed by calculating the overall survival, the disease-free survival and the distant metastasis free survival (DMFS). As expected, T stage was prognostic for overall survival with higher T staged being at more risk for fatal outcome (*p* = 0.012; Figure 4a). The same could be observed for patients with lymph node involvement (*p* < 0.001; Figure 4b) and patients with distant metastases either at primary diagnosis or in the further course of the disease (*p* = 0.03; Figure 4c). There was no influence on overall survival measurable when stratifying for the two main TIL patterns, the presence of TLS or the CD4/CD8 ratio (Figure 4d–f).

**Figure 5:**
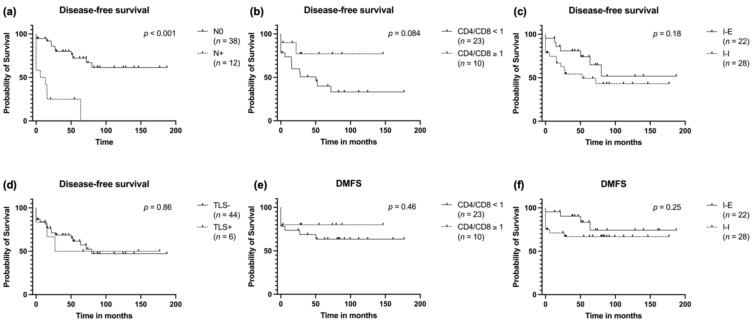
Disease-free survival stratified to (**a**) T stage, (**b**) CD4/CD8 T cell ratio, (**c**) spatial distribution of TIL and (**d**) presence of TLS. Distant metastases free survival stratified to (**e**) CD4/CD8 T cell ratio and (**f**) spatial distribution pattern of TIL.

The differences in recurrence and development of metastases between TIL patterns was reflected in the disease-free survival to a certain extent. This was most evident for the CD4/CD8 ratio, for which a rate of ≥1 seemed to have fewer recurrences, although without reaching significance (Figure 5b). There was no measurable difference depending on TIL pattern or presence of TLS (Figure 5c,d) When looking at the DMFS, again, there was a graphical impression of fewer metastases in patients with a CD4/CD8 ratio ≥ 1 (Figure 5e) and patients with an I-I TIL pattern seemed to be at a higher risk for distant metastases (Figure 5f), although both analyses not being significant in this small cohort.

## 4. Discussion

In this study, we examined the tumor microenvironment of ACC with a focus on spatial patterns of different types of TIL and TLS in serial sections. TLS were only present in a minority of the cases (6/50) and therefore a statistical analysis of any protective role was not possible in a meaningful way. Reports of TLS in cancer were mainly finding a protective role resulting in an improved prognosis for many cancer types (reviewed in [21]). Recently, improved outcomes could also be shown for head and neck squamous cell carcinoma. Especially the presence of germinal center B cells in TLS seemed to be protective, which was even more prominent for TLS within the tumor, but less for TLS outside the tumor [22]. However, it seems that not the mere presence and location of TLS is of prognostic relevance, but more the individual composition of these structures. In a study on lung cancer, it could be shown that TLS with a high number of regulatory T cells (Treg) and a low number of B cells are prognostically worse than low Treg and high B cell TLS [23]. Potentially, the presence of CD21+ B cells (considered to have regulatory functions) was also found to impair the protective role of TLS [24]. Further, the density of HEV seems to play a role and a high density of these vessels in TLS was found to be associated with improved survival. Although, it is not entirely clear if these structures might also not be a path for tumor cells to leave the primary location and form distant metastases [25]. In the present study, we did classify tumors as TLS+ in which these structures were in close proximity to tumor cell nests. Strikingly, the aforementioned factors, which are to be considered protective, such as presence of germinal centers and HEVs, were associated with recurrence in this cohort. Therefore, the role of TLS in ACC might be different, fits to the distinct clinical behavior of this rare entity and supports the ambivalent role of HEVs. Assessing TLS is not without difficulties and many different markers have been proposed to be used in this context [26]. In this study, we have used the most generic markers to find, additionally to spatial TIL patterns, as many TLS as possible. However, there are reports that the number of TLS can be underestimated depending on the methods and markers applied [27]. As this is the first study investigating the rate and role of TIL in combination with TLS in ACC, these results should be pursued and analyzed in larger cohorts. To gain further information on the prognostic role of TLS in this entity it would be beneficial to assess them in more depth and disentangle the immune cell subtypes, especially with regards to B and T regulatory cells.

When disentangling the TME, we found a significantly larger proportion of CD8 T cells compared to CD4 T cells resulting in a low CD4/CD8 ratio. Almost all cases experiencing recurrence had a low ratio or no measurable immune infiltrate. This is somewhat contradictory to previously published studies on the role of TIL and recurrence in ACC. However, interestingly, almost no studies comparing abundance of CD4 and CD8 T cells in the TME of ACC have been published, so far, and most studies focused on assessing CD8 T cells and the role of checkpoint expression. Data from a study on triple negative breast cancer found a significant association of low CD4/CD8 ratio and impaired relapse-free and overall survival [28]. In the present study, CD3 T cells, with the majority being CD8 positive, were found in 56% within the tumor. Another study, focusing on immune checkpoint expression, reported a low CD8+ T cell density in the tumor in 61% and could link this to the development of recurrences. However, the total number of patients (*n* = 36) was smaller than in the present study [9]. This observation was also made by another group when comparing different salivary gland carcinomas. ACC was the one with the least expression of CD8 within the TME. Survival analyses were completed for all tumor entities together due to the small cohort numbers (ACC *n* = 15) [6]. Another group assessed the TME with the help of RNA expression profiles and found, matching our results, fewer CD4 T cells than CD8 T cells in tumors with an immune infiltrate [29]. Finally, a very comprehensive study on the TME of salivary gland tumors showed that ACC had the lowest levels of several types of immune cell RNA signatures compared to other salivary gland carcinoma. The transcriptomic signatures could be verified in a CD3 IHC analysis and low levels of a T cell score were associated with aggressive clinical behavior leading to metastasis and recurrence [8]. Interestingly, none of the above-mentioned studies were able to show a protective role of CD8 T cells in ACC and only few could observe a significant association of low TIL and tumor progression. This leads to the hypothesis that T cells present in the TME of ACC are less capable of a sufficient immune response than in other cancer types. Studies on PD-1 and PD-L1 expression in ACC showed that this mechanism of immune evasion is not common in this disease [6,8,30] and therefore the mechanisms underlying this lack of immunocompetence seem to be independent of the PD-1/PD-L1 axis.

One of the main features of our study is the analysis of the spatial distribution of TIL, which the aforementioned studies are mostly lacking. To divide cases according to the dominant infiltration pattern, we distributed them into two main types, infiltrated-inflamed and infiltrated-excluded. These categories have been established in a review by Binnewies et al. in 2018. Therein, I-I tumors are described as immunologically hot versus I-E being cold tumors with immune cells only located at the invasive margins, but not in the tumor core [18]. However, there was no obvious association between the TIL patterns and clinical outcome detectable in our study. This may well be due to the fact that these established patterns are not relevant in ACC as it is a non-immunogenic cancer, which is supported by the failure of various immunotherapy trials [11,12,13,14]. Another reason for the lack of protective immunity could be the actual composition of T cell subsets. It is now a well-established concept that protective immunity is dependent on T resident memory cells (T_RM_) [31,32]. Therefore, a more comprehensive analysis would be needed, which incorporates spatial data and profound transcriptomic or proteomic profiling. These techniques are now available with the introduction of imaging mass cytometry or spatial gene expression, and it would make sense to apply them to this rare disease as well.

Finally, in survival analyses of the cohorts, we could not find any immune infiltrate related markers for prognosis. A possible selection bias could be ruled out when looking at the significant association of higher T stages with impaired overall survival as expected. This was also reported by Chen et al. in early 2021. They found very low TIL abundance in half of the cases and no influence on survival [33]. However, one limitation of our study is the follow-up period. Since metastases can occur up to 10 years after primary diagnosis, we may have missed some recurrences, as only 18 % of patients reached this period. On the other hand, over 50% of patients were followed up for more than 5 years, which we believe is acceptable given the retrospective nature of the study.

## 5. Conclusions

Spatial analysis of TIL within the TME of ACC revealed a fairly even distribution of cases into an infiltrated-excluded and an infiltrated-inflamed type with very few carcinomas containing TLS. The patterns had no clear influence on prognosis, but most cases with recurrence showed either a low CD4/CD8 ratio in I-I tumors or an I-E pattern. This adds to the understanding of ACC being a low immunogenic tumor in which the effect of immunotherapies seems to be limited.

## Figures and Tables

**Table 1:** Patient characteristics at initial diagnosis and treatment parameters stratified by TIL patterns.

Clinical Parameter	Infiltrated-Excluded (*n* = 22)	Infiltrated-Inflamed (*n* = 28)	*p*-Value ^1^
**Gender**			0.95
Male	10 (43.5%)	13 (56.5%)	
Female	12 (44.4%)	15 (55.6%)	
**Age (average in years)**	55.23	59.68	0.34
**Tumor site**			0.21
Submandibular gland	4 (23.5%)	13 (76.5%)	
Parotid gland	6 (66.7%)	3 (33.3%)	
Paranasal sinus	1 (16.7%)	5 (83.3%)	
Base of tongue	3 (60%)	2 (40%)	
Nasal cavity	2 (66.7%)	1 (33.3%)	
Hard palate	2 (100%)	0 (0%)	
Sublingual gland	1 (50%)	1 (50%)	
Soft palate	1 (50%)	1 (50%)	
Nasopharynx	0 (0%)	1 (100%)	
Oral cavity	0 (0%)	1 (100%)	
Larynx	1 (100%)	0 (0%)	
External auditory meatus	1 (100%)	0 (0%)	
**T-classification**			0.23
T1–2	14 (51.9%)	13 (48.1%)	
T3–4	8 (34.8%)	15 (65.2%)	
**N-classification**			0.64
N0	18 (47.4%)	20 (52.6%)	
N1	1 (25%)	3 (75%)	
N2–3	3 (37.5%)	5 (62.5%)	
**M-classification**			0.06
M0	21 (51.2%)	20 (48.8%)	
M1	1 (12.5%)	7 (87.5%)	
**Stage**			0.03
I-IVA	20 (52.6%)	18 (47.4%)	
IVB-IVC	2 (16.7%)	10 (83.3%)	
**Primary therapy**			0.03
Surgery alone	12 (70.6%)	5 (29.4%)	
Surgery + adj. RT	8 (29.6%)	19 (70.4%)	
RT	2 (40%)	3 (60%)	
**R-Status ^2^**			0.27
R0	11 (52.4%)	10 (47.6%)	
R0 after re-resection	5 (55.6%)	4 (44.4%)	
R1	3 (25%)	9 (75%)	
R2	1 (100%)	0 (0%)	
**Pn ^2^**			0.47
Pn0	10 (50%)	10 (50%)	
Pn1	9 (39%)	14 (60.9%)	

^1^ Comparison was computed either with Chi^2^-Test or with Fisher’s Exact test according to expected cell counts. ^2^ Information only available for patients treated with surgery.

**Table 2:** TIL patterns and presence/development of distant metastases and recurrences.

Patterns of Failure	Infiltrated-Inflamed	Infiltrated-Excluded	Presence of TLS ^†^	CD4/CD8 Ratio < 1 *
Distant metastasis at initial diagnosis (8/50)	7 (87.5%)	1 (12.5%)	1 (12.5%)	5/7 (71.4%)
Recurrence (15/50)	7/15 (46.7%)	8/15 (53.3%)	2/15 (13.3%)	9/10 (90%)
Local recurrence (11/50)	7/11 (63.3%)	4/11 (36.4%)	1/11 (9.3%)	7/8 (87.5%)
Distant recurrence (6/50)	2/6 (33.3%)	4/6 (66.7%)	1/6 (16.7%)	3/3 (100%)

^†^ TLS were only present in infiltrated-inflamed tumors, percentage is of number of recurrences. * Calculated for cases with measurable immune infiltrate (*n* = 32).

## Data Availability

The data presented in this study are available on request from the corresponding author. The data are not publicly available due to ethical restrictions.

## Supplementary Materials

The following supporting information can be downloaded at: https://www.mdpi.com/article/10.3390/cancers14061383/s1, Table S1: Antibodies, Table S2: Morphological features of TLS and clinical data.
