# Supplementary material for: Patterns of Tumor Infiltrating Lymphocytes in Adenoid Cystic Carcinoma of the Head and Neck
Source: Cancers (Basel). 2022 Mar 8;14(6):1383. doi: 10.3390/cancers14061383 (PMC8946094; doi:10.3390/cancers14061383)
Supplement: Supplementary file 1 [file cancers-14-01383-s001.zip › cancers-1602050-supplementary.pdf]

Supplementary Materials: Patterns of Tumor Infiltrating Lymphocytes in Adenoid Cystic Carcinoma of the Head and Neck

Johannes Doescher, Moritz Meyer, Christoph Arolt, Alexander Quaas, Jens Peter Klußmann, Philipp Wolber, Agnes Bankfalvi, Hans-Ulrich Schildhaus, Tobias Bastian, Stephan Lang, Simon Laban, Patrick J. Schuler, Cornelia Brunner, Thomas K. Hoffmann and Stephanie E. Weissinger

Table S1. Antibodies.

Antibody	Company	Clone/Cat.No	Dilution	Pretreatment	Staining Pattern
CD3	Agilent/Dako, USA	poly, CD3	rtu	low	mem
CD4	Agilent/Dako, USA	m, 4B12	rtu	high	mem
CD8	Agilent/Dako, USA	m, C8/144B	rtu	high	mem
CD20	Agilent/Dako, USA	m, L26	rtu	high	mem

Abbreviations: m, monoclonal; mem, membranous; poly, polyclonal; rtu, ready-to-use.

Table S2. Morphological features of TLS and clinical data.

Case	Tumor Location	Density of HEV	Presence of GC	Recurrence
1	Submandibular gland	None	No	No
2	Soft palate	Low	Yes	Yes
3	Base of tongue	None	No	No
4	Submandibular gland	None	No	No
5	Sublingual gland	None	No	No
6	Submandibular gland	High	Yes	Yes

Abbreviations: HEV, high endothelial venule; GC, germinal center.
